# Generating synthetic brain PET images of synaptic density based on MR T1 images using deep learning

**DOI:** 10.1186/s40658-025-00744-5

**Published:** 2025-03-31

**Authors:** Xinyuan Zheng, Patrick Worhunsky, Qiong Liu, Xueqi Guo, Xiongchao Chen, Heng Sun, Jiazhen Zhang, Takuya Toyonaga, Adam P. Mecca, Ryan S. O’Dell, Christopher H. van Dyck, Gustavo A. Angarita, Kelly Cosgrove, Deepak D’Souza, David Matuskey, Irina Esterlis, Richard E. Carson, Rajiv Radhakrishnan, Chi Liu

**Affiliations:** 1 Department of Biomedical Engineering, Yale University, New Haven, CT USA; 2 Department of Psychiatry, Yale University, New Haven, CT USA; 3 Department of Radiology and Biomedical Imaging, Yale University, New Haven, CT USA; 4 Department of Psychology, Yale University, New Haven, CT USA

**Keywords:** PET, Neuroimaging, [^11^C]UCB-J, SV2A, Medical imaging synthesis, Deep learning

## Abstract

**Purpose:**

Synaptic vesicle glycoprotein 2 A (SV2A) in human brains is an important biomarker of synaptic loss associated with several neurological disorders. However, SV2A tracers, such as [^11^C]UCB-J, are less available in practice due to constrains such as cost, radiation exposure and onsite cyclotron. We therefore aim to generate synthetic [^11^C]UCB-J PET images based on MRI in this study.

**Methods:**

We implemented a convolution-based 3D encoder-decoder to predict [^11^C]UCB-J SV2A PET images. A total of 160 participants who underwent both MRI and [^11^C]UCB-J PET imaging, including individuals with schizophrenia, cannabis use disorder, Alzheimer’s disease, were used in this study. The model was trained on pairs of T1-weighted MRI and [^11^C]UCB-J distribution volume ratio images, and tested through a 10-fold cross-validation process. The image translation accuracy was evaluated based on the mean squared error, structural similarity index, percentage bias and Pearson’s correlation coefficient between the ground truth and the predicted images. Additionally, we assessed the prediction accuracy of selected regions of interest (ROIs) crucial for brain disorders to evaluate our results.

**Results:**

The generated SV2A PET images are visually similar to the ground truth in terms of contrast and tracer distribution, quantitatively with low bias (< 2%) and high similarity (> 0.9). Across all diagnostic categories and ROIs, including the hippocampus, frontal, occipital, parietal, and temporal regions, the synthetic SV2A PET images exhibit an average bias of less than 5% compared to the ground truth. The model also demonstrates a capacity for noise reduction, producing images of higher quality compared to the low-dose scans.

**Conclusion:**

We conclude that it is feasible to generate robust SV2A PET images with promising accuracy from MRI via a data-driven approach.

**Supplementary Information:**

The online version contains supplementary material available at 10.1186/s40658-025-00744-5.

## Introduction

Positron Emission Tomography (PET) imaging can provide functional and molecular information about the brain and is increasingly integrated into the research of various brain disorders. [^11^C]UCB-J is a PET tracer for imaging the synaptic vesicle protein 2 A (SV2A) that is ubiquitously expressed in the synapses in human brains and has garnered interest as an important biomarker of synaptic loss in various disorders including schizophrenia, Alzheimer’s disease, and substance use disorders [1]. However, the utilization and accessibility of PET imaging with SV2A tracers are constrained by factors including risks due to exposure to radiation (which limits its use in pregnancy and adolescents, as well as the number of scans that can be performed in a year given federal limits on annual radiation exposure), increased cost, need for an onsite cyclotron, the requirement of specialized scanners, and need for expertise in data acquisition and processing. It is therefore of interest to investigate the feasibility of generating synthetic SV2A PET images using deep learning-based approaches from Magnetic Resonance Imaging (MRI), which is more easily accessible, less expensive, and free of ionizing radiation. In this study, we investigated this feasibility using three prototypical neuropsychiatric disorders as a sample population, including schizophrenia (SZ), relevant to evaluation of synaptic density in adolescence; Alzheimer’s disease (AD), relevant to limiting total radiation exposure; and cannabis use disorder (CUD), relevant to evaluation of synaptic density during pregnancy.

Schizophrenia is a chronic, disabling psychiatric disorder which incurs an annual cost of $343 billion in the United States, and is a leading cause of disability worldwide [2]. Synaptic density loss is the most consistently reported finding in postmortem studies [3] and the genetic risk for the disorder converges on synaptic proteins. SV2A PET imaging, using [^11^C]UCB-J, in schizophrenia has shown that synaptic density is lower in chronic schizophrenia (replicated by two independent research groups) [4, 5] and may be present in early stages of the disorder [6, 7]. The disorder typically emerges in the teen years, and adolescence is hypothesized to be a critical period of neurodevelopment relevant to this disorder. Hence, it is of great interest to examine synaptic density in adolescence and teenagers who are at-risk or in early stages of the disorder.

Alzheimer’s disease is a major neurodegenerative disorder with a prevalence of approximately 10% among people aged 65 years and older [8]. Synaptic loss is a significant neuropathological finding in the disorder [9]. PET imaging for amyloid and tau are FDA-approved for the evaluation of Alzheimer’s disease. Patients with Alzheimer’s disease may undergo FDG PET to establish the diagnosis when there is a need to differentiate it from other causes of dementia [10]. SV2A PET imaging studies have consistently reported lower synaptic density in Alzheimer’s disease [11, 12]. The ability to generate SV2A PET images from MRI images can lower the total radiation exposure in this population and enable serial monitoring of response to treatment.

Cannabis use is becoming increasingly prevalent in the United States given the change in the legal status of cannabis in different states that have legalized “medical cannabis” and the consequent decrease in the perception of harm from cannabis among adolescents [13]. Cannabis is also increasingly being used by pregnant women to treat nausea in pregnancy [14]. SV2A PET imaging using [^11^C]UCB-J in cannabis use disorder has shown lower hippocampal and prefrontal cortical synaptic density in cannabis use disorder [15, 16], but the effects of cannabis use on synaptic density during pregnancy have not been examined as the risks of radiation during pregnancy with PET imaging make such a study prohibitive.

In recent years, there has been a rapid evolution in image analysis and processing systems driven by the adoption of deep learning methods. These models have demonstrated remarkable success in helping with various medical image tasks including classification, segmentation and detection —tasks traditionally relied on the expertise of trained physicians [17] and have gained popularity as a powerful tool in medical image synthesis and translation [18–20]. Particularly, a large amount of work on variants of CNNs and GANs has been conducted in cross-modality image-to-image synthesis using deep learning methods [21]. Jyoti and Zhang [22] trained a GAN to generate brain PET images, in order to help data augmentation. Kearney et al. [23] trained a cycleGAN for brain MR-to-CT image translation. Fard et al. [24] also proposed using a cGAN to translate brain PET and MRI scans to SPECT images. Osman and Tamam [18] used U-net for brain MRI synthesis across T1, T2 and FLAIR. Shi et al. [25] proposed using a U-net with modified loss function for PET attenuation map generation. Wang et al. [26] employed a U-net for the conversion from [^18^F]FDG to [^11^C]UCB-J for brain PET images. Hu et al. [27] employed a bidirectional mapping GAN for brain MRI to [^18^F]FDG PET synthesis. Zhang et al. [28] used a spatial adaptive and transformer fusion network (STFNet) for the denoising of low-count [^18^F]FDG PET with MRI. Xie et al. [29] used a 2D conditional diffusion model to generate [^18^F]FDG PET from high-field or ultra-high-field MRI data. Jiang et al. [30] developed a 2D latent diffusion model to generate Tau PET from textual descriptions and MRI. Furthermore, research indicates that changes in synaptic density can affect brain energetics and consequently MRI signals [31]. While [^11^C]UCB-J PET measures synaptic density and structural MRI does not, this putative relationship between MRI estimates of gray matter morphometry and synaptic density has been identified and supported, providing a theoretical foundation for this project. As a result, in this research we investigated if it is feasible to translate MR T1-weighted images into SV2A PET images. We addressed the task of generating synthetic SV2A PET for healthy participants as well as participants with SZ, AD and CUD. As GANs are challenging to train and prone to mode collapse [32] and as diffusion-based models (DMs) tend to produce hallucinations, here we developed a 3D encoder-decoder to create a fast and stable one-step inference method. We managed to generate 3D brain SV2A PET images of diagnostic quality and quantified our predictive accuracy for selected regions of interest (ROIs) crucial for understanding brain disorders.

## Materials and methods

### Datasets

As shown in Table 1, we included 160 participants who underwent both MRI and [^11^C]UCB-J PET scans. This cohort includes 24 participants with SZ, 34 participants with AD, 19 participants with CUD, and 83 healthy (HC) volunteers who participated in research protocols approved by Yale Institutional Review Board (IRB). The mean injected activity was 15.71 mCi for [^11^C]UCB-J. The MR and PET scans were acquired at the Yale MR Research Center and the Yale PET Center respectively. [^11^C]UCB-J radiosynthesis and PET imaging procedures were identical across primary studies and have been reported previously [4, 15, 33]. Briefly, [^11^C]UCB-J was administered as an intravenous bolus injection over one minute and up to 120 min of dynamic PET data were acquired on an HRRT system (Siemens/CTI, Knoxville, TN, USA). Blood sampling was performed during PET scans using arterial catheters for metabolite analysis, a transmission scan was obtained for attenuation correction, and head motion was measured with a Polaris Vicra optical tracking system (NDI Systems, Waterloo, Canada). PET listmode data were reconstructed into volumes of 256 × 256 × 207 with a voxel size of 1.219 × 1.219 × 1.231 mm^3^ with normalization and corrections for attenuation, scatters, randoms, deadtime, and motion using a listmode reconstruction platform [34]. Parametric images of [^11^C]UCB-J non-displaceable binding potential (*BP*_ND_) were estimated using a reference tissue model (SRTM2) with a centrum semiovale reference region.

**Table 1:** Demographics of the dataset

Demographics and Injection Information	*Dataset (n = 160)*
Age, mean (SD) [min, max], years	49.6 (19.6) [19.0, 84.5]
Sex, Male: Female	97:63
Diagnosis, SZ: AD: CUD: HC	24:34:19:83
[^11^C]UCB-J Injection Activity, mean (SD), mCi	15.71 (4.32)

T1-weighted (T1W) MRI images were collected on a Siemens 3T MAGNETOM (TIM Trio and PrismaFit) imaging systems (Siemens, Erlangen, Germany) using standard magnetization-prepared rapid-acquisition gradient-echo (MPRAGE) sequences (Table S1). Whole-head T1-weigthed images were processed using the Computational Anatomy Toolbox 12 (CAT12; version 12.8; Structural Brain Imaging Group, University of Jena) for SPM12 (Wellcome Centre for Human Neuroimaging, London, UK) following the default workflow including spatial adaptive non-local denoising, bias-field correction, initial tissue segmentation, regional parcellation, and refined locally-adaptive segmentation that includes intensity transformation and partial volume estimation [35].

Notably, these steps include image processing techniques similar to multi-site harmonization techniques (e.g., bias field correction, denoising, and intensity normalization [36]) that typically require substantially larger intra-site samples than the current study included across the different magnets and sequences [37]. Furthermore, the images included were all collected at the same field strength (i.e., 3T) using scanners from the same manufacturer (i.e., Siemens) and using nearly identical image resolution parameters (i.e., 1 × 1 × 1 mm ± 0.05 mm in-plane, and ± 0.2 mm in slice thickness), minimizing primary sources of intra-site variability. Examination of the voxel value distributions demonstrated that any potential differences between images from the different scanners and sequences were effectively mitigated by the processing methods employed in CAT12.

Specifically, each participant’s [^11^C]UCB-J *BP*_ND_ image was aligned to their processed T1W image using the co-registration function in SPM12. The estimated gray-matter, white-matter, and cerebral spinal fluid tissue segments from CAT12 were combined to generate a brain mask that was applied to the [^11^C]UCB-J *BP*_ND_ image and the T1W image in participant native space, and the masked T1w image was resampled in SPM12 to the voxel dimensions of the PET images (1.219 × 1.219 × 1.231 mm^3^, image size 256 × 256 × 207). The cerebellum and all other regions of interest used in subsequent analyses were defined using the CAT12-estimated inverse non-linear transformation applied to the automated anatomical labelling atlas 3 (aal3) [38] for each participant. Consistent with prior reports generating synthetic [^11^C]UCB-J PET data from [^18^F]FDG images, parametric [^11^C]UCB-J images of distribution volume ratio (DVR) were generated with the cerebellum (Cb) as the reference region, calculated as DVR = (*BP*_ND_+1) / (*BP*_ND_[Cb] + 1) [26].

### Deep learning model architecture

We employed a symmetric 3D encoder-decoder with convolutional layers for the synthetic SV2A PET generation task. This framework consists of two components: the encoder extracts information from the input, and the decoder up-samples the encoded representations to generate the results based on compressed information. The loss function guides the learning process by quantifying the difference between the input and the output image generated by the decoder. This framework design offers a simple training and fast inference, leveraging convolutional filters to effectively extract information through hierarchical feature learning. In our study, the MR images are used as the input layer of the encoder, and the synthetic PET DVR images are the output of the decoder and subsequently compared with the ground truth DVR images. The model was trained on paired images from the same participant.

**Fig. 1:**
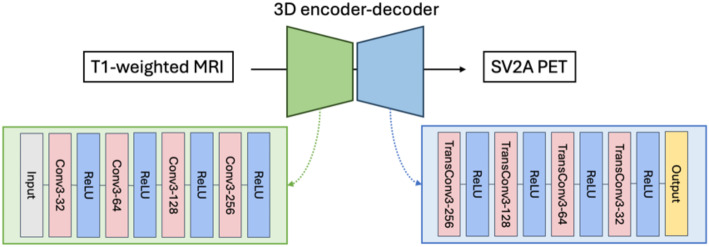
Illustration of the proposed encoder-decoder network. The encoder-decoder has a symmetric design, with convolutional layers and transposed convolutional layers of kernel size 3 × 3 × 3 employed in the encoder and decoder, respectively. The number of channels were doubled at each step in the encoder and halved at each step in the decoder

The encoder has a series of 4 convolutional layers with an incrementally increasing number of filters to down sample and extract features from the inputs, while the decoder employed a progressively decreasing number of filters across its 4 transposed convolutional layers to up sample the features to the input dimension, as shown in Fig. 1. In the initial layer, 32 features were extracted by 3 × 3 × 3 3D convolution kernels. Subsequently, the number of features were doubled during each down-sampling step in the encoder and halved during each up-sampling step in the decoder. This results in an encoder with filter count of 32, 64, 128, and 256, and a decoder with filter count of 256, 128, 64, and 32. A convolutional layer with filter number of 1 was used as the output layer. All layers used the same kernel size of 3 × 3 × 3. ReLU activation functions are applied after each convolutional operation. Valid padding was applied to each layer.

### Experimental setup

The training and testing were carried out using NVIDIA A40 GPUs. For training, preprocessed MR T1-weighted images and PET DVR images are randomly cropped into 64 × 64 × 64 patches to be fed into the model. As a common practice [19, 26], the patch size was selected to strike a balance, ensuring it was large enough for the models to capture patterns effectively while maintaining computational efficiency. The batch size was set to 4. Each epoch contained 100 steps, resulting in 400 patches used in each epoch. 150 epochs were run to achieve the convergence of the L2 loss function. The Adam optimizer was employed with an initial learning rate of 0.001, which exponentially decayed by a factor of 0.99 every 100 steps. During testing, we configured the input layer and the input patch size to be 240 × 240 × 160, ensuring comprehensive coverage of the entire brain of each participant without any overlaps to avoid artifacts at the edge of the patches, while staying within the limits of the maximum allowable GPU memory.

The participant data used for training, validation and testing the model were randomly sampled from the SZ, AD, CUD, and HC groups to construct datasets with a balanced diagnostic ratio. We employed 10-fold cross-validation to test all the participants and evaluate the performance of our model. Specifically, for each fold 130 participants were allocated for training, 7 participants were reserved for validation, and 23 participants were set aside for testing. There were 5 participant datasets with low injection dose (< 5 mCi) showing high-noise DVR images, and they were included only in the testing phase for each fold and evaluated repeatedly but were not included in training.

### Image quality metrics

The image translation quality was assessed in terms of mean squared error (MSE), structural similarity index (SSIM), percentage bias, and Pearson’s correlation coefficient ($\:\rho\:$) between the ground truth PET images and the predicted SV2A images. Mean percentage bias of various brain ROIs was also computed. The metrics were calculated as follows:

• MSE

$$\:MSE=\frac{1}{n}{\sum\:}_{n}^{i=1}{\left|\left|{I}_{x}-{I}_{y}\right|\right|}^{2}$$

where *I*_*y*_ is the predicted image from the MR T1-weighted image and *I*_x_ is the ground truth image.

• SSIM

$$\:SSIM=\frac{\left(2{\mu\:}_{x}{\mu\:}_{y}+{c}_{1}\right)\left(2{\sigma\:}_{xy}+{c}_{2}\right)}{\left({\mu\:}_{x}^{2}+{\mu\:}_{y}^{2}+{c}_{1}\right)\left({\sigma\:}_{x}^{2}+{\sigma\:}_{y}^{2}+{c}_{2}\right)}$$

where *µ*_*x*_ and *µ*_*y*_ indicate the mean of the voxel values from the real and predicted PET SV2A images, *σ*_*x*_ and *σ*_*y*_ are the standard deviation of the real and predicted volume voxels and *σ*_*xy*_ represents the covariance of the real and predicted volume voxels. *c*_*1*_ and *c*_*2*_ are regularization constants chosen proportional to the dynamic range of the voxel values of the images to avoid numerical instability for image regions where the local mean or standard deviation is close to zero.

• Bias

$$\:Percentage\:Bia{s}_{R}=\frac{{\mu\:}_{{x}_{R}}-{\mu\:}_{{y}_{R}}}{{\mu\:}_{{x}_{R}}}$$

where $\:{\mu\:}_{{x}_{R}}$ and $\:{\mu\:}_{{y}_{R}}$ denote the mean voxel values within a region R, which could be the whole image or certain regions of interest (ROIs).

• Pearson’s correlation

$$\:\rho\:=\frac{Cov\left({I}_{x},{I}_{y}\right)}{{\sigma\:}_{x}{\sigma\:}_{y}}$$

where *I*_x_ and *I*_*y*_ are the true and predicted volumes, and *σ*_*x*_ and *σ*_*y*_ are the standard deviations of the real and predicted volume.

## Results

**Fig. 2:**
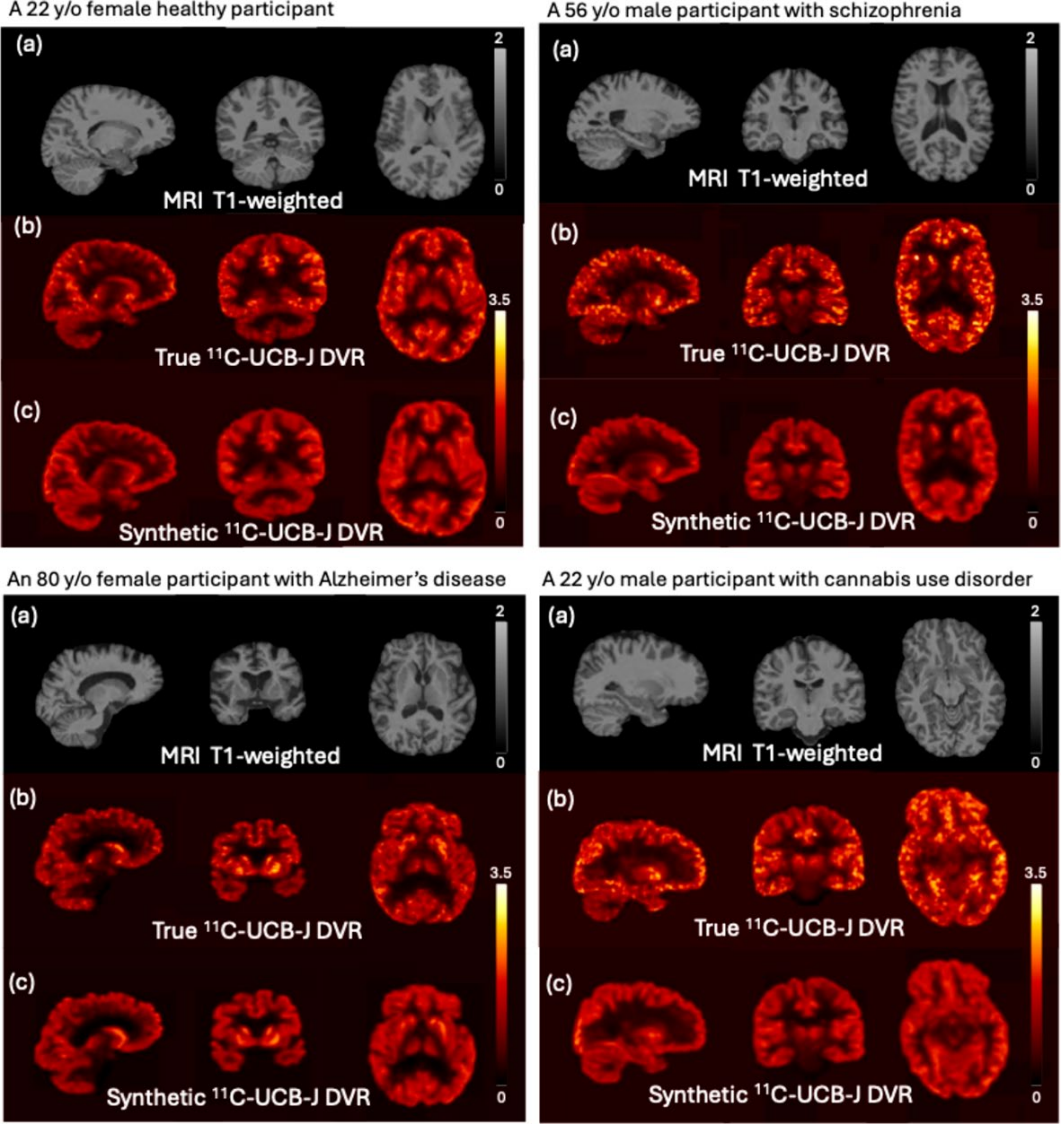
Four sample results from each diagnostic categories: HC, SZ, AD and CUD. (**a**) T1-weighted MRI, input of the model; (**b**) [^11^C]UCB-J PET DVR image, ground truth; (**c**) Synthetic [^11^C]UCB-J DVR image, output of the model

**Fig. 3:**
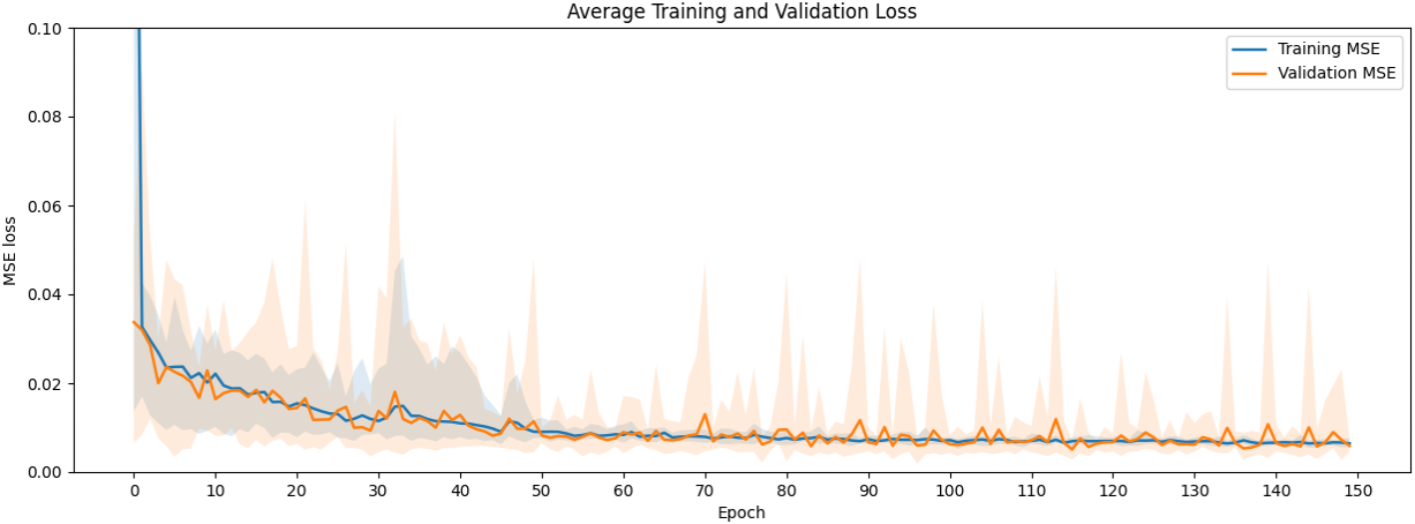
Average training and validation loss. The solid lines denote the mean training and validation MSE over 150 epochs across the ten-fold cross validation, while the shaded areas illustrate the range of the loss

Figure 2 shows sample synthetic PET images and the corresponding ground truth PET image. The synthetic PET images are highly consistent with the true images in terms of tracer distribution and contrast for both healthy volunteers and participants with various diseases. The synthetic PET images have lower noise levels but are smoother than the true PET images. Figure 3 shows the average training and validation MSE loss over the epochs across the 10 folds, indicating the convergency of the model after 100 epochs.

As shown in Tables 2 and 3, the quantitative results were consistent with the visual qualitative observation. The whole image mean MSE of the network was 0.0062 ± 0.0071 across the HC group, 0.0072 ± 0.0037 across the SZ group, 0.0067 ± 0.0045 across the AD group and 0.0054 ± 0.0029 across the CUD group. The mean SSIM was 0.90 ± 0.05 across the participants in the HC group, 0.89 ± 0.05 across the SZ group, 0.91 ± 0.06 across the AD group, and 0.89 ± 0.05 across the CUD group, showing that the perceived change in structural information between the synthetic and gold standard images is small. The whole image mean percentage bias between the generated images and the ground truth [^11^C]UCB-J images was − 1.55% ± 12.47% across the HC group, 1.54% ± 12.25% across the SZ group, -4.47% ± 16.57% across the AD group and − 1.46% ± 10.83% across the CUD group, indicating the predicted synthetic PET achieved low absolute deviation. Additionally, we conducted a leave-one-disease-out experiment: training the model on three diseases while testing it on the fourth to assess its generalizability. The results are shown in Table 4. For HC, SZ and CUD groups, the model achieved a bias of less than 2% and predicted relatively well when generalizing. Meanwhile, for the AD group, as it exhibits distinct neurodegenerative patterns, it is not surprising to observe that the average prediction bias became around − 6.6% for AD when the model is trained solely on HC, SZ and CUD.

**Table 2:** 10-fold cross-validated prediction performance

Mean ± St.Dev.	BIAS	MSE	SSIM	$\:\rho\:$
All	-1.695% ± 13.246%	0.0064 ± 0.0058	0.9006 ± 0.0524	0.9602 ± 0.0140
*HC*	-1.550% ± 12.473%	0.0062 ± 0.0071	0.9008 ± 0.0499	0.9637 ± 0.0140
SZ	1.538% ± 12.252%	0.0072 ± 0.0037	0.8924 ± 0.0537	0.9521 ± 0.0146
AD	-4.466% ± 16.571%	0.0067 ± 0.0045	0.9112 ± 0.0562	0.9541 ± 0.0114
CUD	-1.456% ± 10.830%	0.0054 ± 0.0029	0.8915 ± 0.0547	0.9659 ± 0.0084

**Table 3:** 10-fold cross-validated prediction performance for low-dose participants

Low-dose participants	BIAS	MSE	SSIM	$\:\rho\:$
Mean	0.069%	0.012	0.8136	0.9103
St. Dev.	11.093%	0.004	0.0356	0.0283

**Table 4:** Leave-one-disease-out model performance test result

Mean ± St.Dev.	BIAS	MSE	SSIM	$\:\rho\:$
*HC*	-1.754% ± 12.28%	0.0061 ± 0.0074	0.9265 ± 0.0427	0.9643 ± 0.0126
SZ	1.204% ± 10.83%	0.0071 ± 0.0029	0.8931 ± 0.0494	0.9537 ± 0.0083
AD	-6.633% ± 16.21%	0.0065 ± 0.0043	0.9140 ± 0.0503	0.9575 ± 0.0109
CUD	0.196% ± 12.30%	0.0055 ± 0.0036	0.8928 ± 0.0528	0.9648 ± 0.0146

**Fig. 4:**
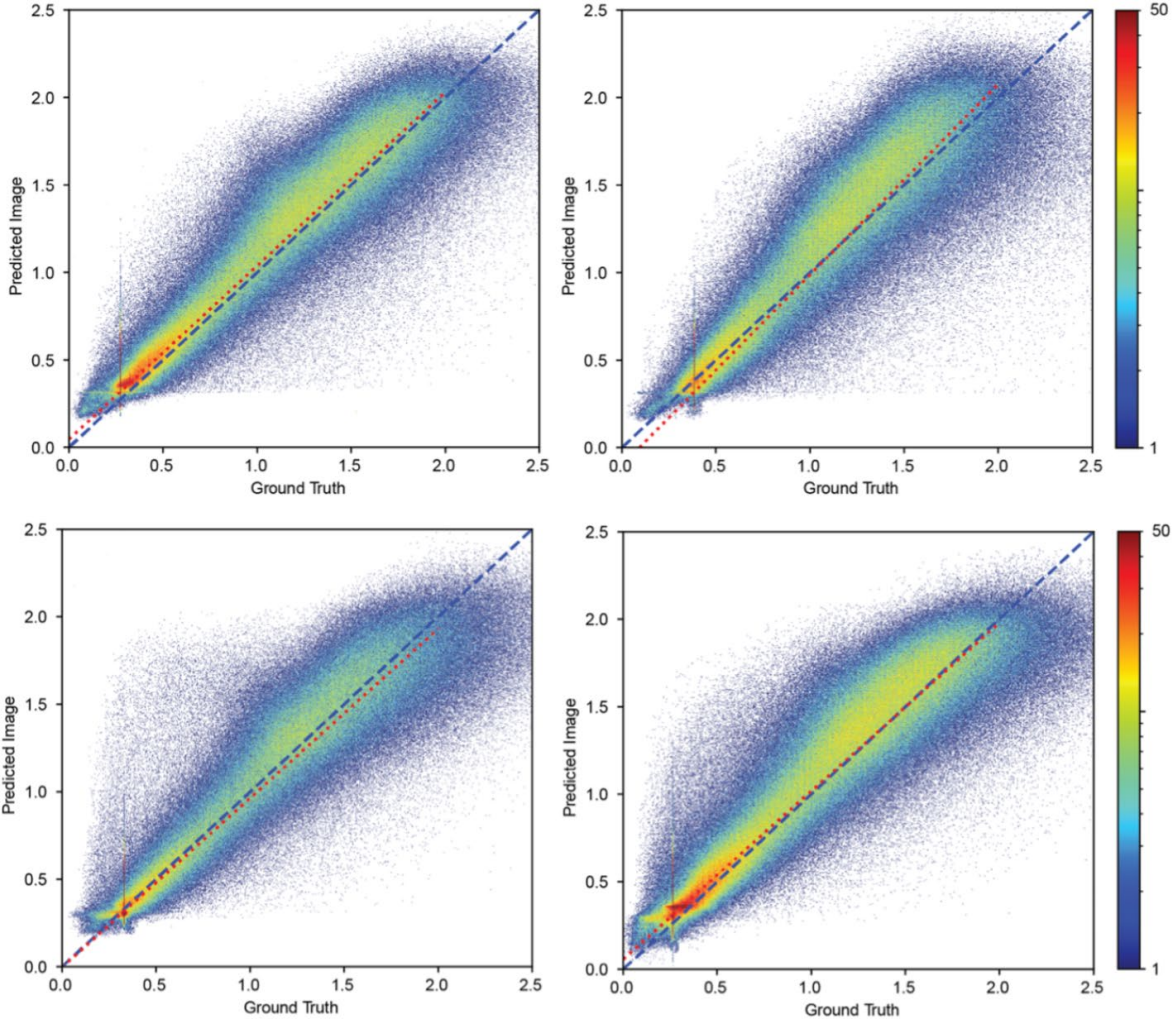
Joint histograms with linear fitting between the predicted voxel values and the true voxel values for the sample participants from each diagnostic categories: HC, SZ, AD and CUD

Consistency analysis of histogram distribution was performed voxel-by-voxel between the true [^11^C]UCB-J PET image and the predicted [^11^C]UCB-J PET image. The correlation plot between the predicted image voxel values and true image voxel values with linear fitting is shown in Fig. 4. The average Pearson’s correlation for normal-dose participants was 0.96 ± 0.01 between the voxel values of the predicted and ground truth image across the participants in the HC group, 0.95 ± 0.01 across the SZ group, 0.95 ± 0.01 across the AD group, and 0.97 ± 0.01 across the CUD group.

**Fig. 5:**
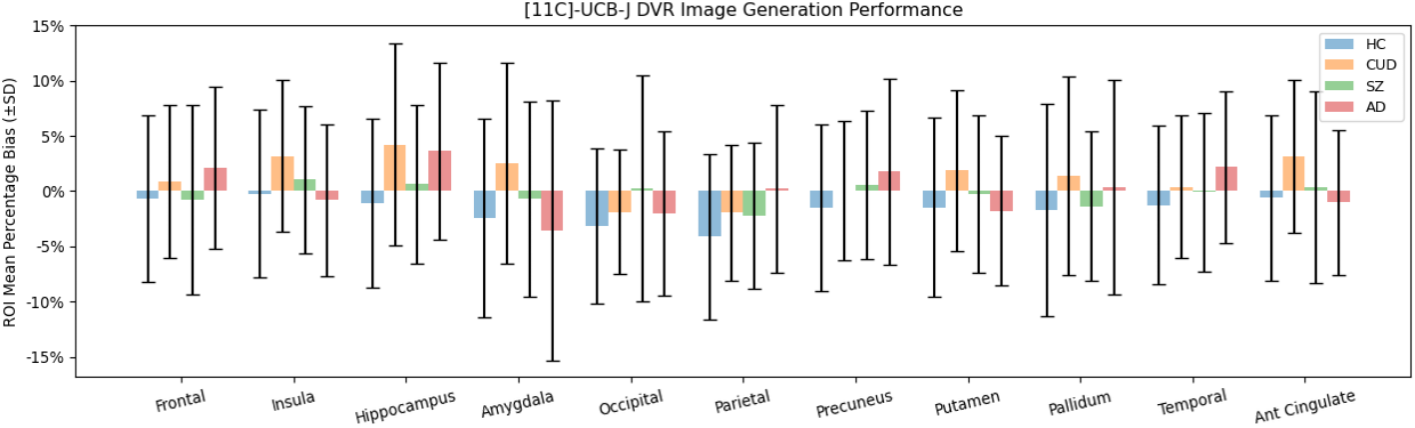
Average (± SD) percentage bias between the real PET scans and the model-generated images for different ROIs across all diagnostic categories

The mean and standard deviation of percentage bias and SSIM between the prediction and ground truth of all selected ROIs for all normal dose participants are shown in Tables 5 and 6. Across all diagnostic categories for all examined ROIs, including the hippocampus, frontal, occipital, parietal, and temporal regions, the generated images demonstrated an average bias of less than 5% from the ground truth [^11^C]UCB-J images (Fig. 5). Additionally, we found that the predicted images for participants with Alzheimer’s disease had the lowest performance across all diagnostic types in terms of the percentage bias. We performed 2-sample t-tests for the ROI voxel values and found that the differences between AD participants and healthy volunteers in certain regions (e.g., for hippocampus, *p* = 0.00 in the true images and *p* = 0.00 in the generated images) are still significant in the generated images. Similarly, for participants with low-dose injection, the ROI analysis results were shown in Table 7. As expected, the average percentage bias for each ROI for the low-dose images was significantly higher than the normal-dose ones, and the SSIM for each ROI between the low-dose PET image and the synthetic image was also lower.

**Table 5:** 10-fold cross-validated mean (± SD) percentage bias between model prediction and ground truth for ROIs

	Frontal	Insula	Hippocampus	Amygdala	Occipital	Parietal	Precuneus	Putamen	Pallidum	Temporal	Ant. Cingulate
*All*	0.08%±7.71%	0.25%±7.32%	0.81%±8.15%	-1.82%±9.80%	-2.24%±7.62%	-2.67%±7.45%	-0.31%±7.62%	-0.96%±7.67%	-0.84%±9.25%	-0.16%±7.14%	-0.11%±7.54%
*HC*	-0.68%±7.58%	-0.22%±7.60%	-1.07%±7.64%	-2.40%±8.98%	-3.12%±7.02%	-4.13%±7.50%	-1.49%±7.56%	-1.47%±8.09%	-1.68%±9.59%	-1.27%±7.15%	-0.62%±7.50%
*SZ*	-0.81%±8.57%	1.05%±6.66%	0.63%±7.15%	-0.72%±8.79%	0.23%±10.21%	-2.25%±6.59%	0.56%±6.72%	-0.29%±7.10%	-1.36%±6.80%	-0.11%±7.14%	0.39%±8.66%
*AD*	2.13%±7.36%	-0.80%±6.87%	3.64%±8.01%	-3.57%±11.80%	-1.99%±7.43%	0.20%±7.63%	1.76%±8.44%	-1.76%±6.79%	0.33%±9.70%	2.19%±6.89%	-1.02%±6.56%
*CUD*	0.84%±6.92%	3.19%±6.83%	4.18%±9.14%	2.49%±9.09%	-1.91%±5.63%	-1.97%±6.11%	0.03%±6.29%	1.87%±7.28%	1.42%±8.99%	0.40%±6.43%	3.15%±6.90%

**Table 6:** 10-fold cross-validated mean (± SD) SSIM between model prediction and ground truth for ROIs

	Frontal	Insula	Hippocampus	Amygdala	Occipital	Parietal	Precuneus	Putamen	Pallidum	Temporal	Ant. Cingulate
*All*	0.7382 ± 0.0620	0.6119 ± 0.0532	0.5928 ± 0.0574	0.5423 ± 0.0715	0.6532 ± 0.0580	0.6520 ± 0.0560	0.6747 ± 0.0593	0.6233 ± 0.0687	0.7476 ± 0.0740	0.6233 ± 0.0518	0.7040 ± 0.0551
*HC*	0.7513 ± 0.0565	0.6186 ± 0.0492	0.5980 ± 0.0547	0.5426 ± 0.0733	0.6688 ± 0.0539	0.6622 ± 0.0536	0.6889 ± 0.0532	0.6297 ± 0.0680	0.7513 ± 0.0740	0.6293 ± 0.0497	0.7150 ± 0.0462
*SZ*	0.7136 ± 0.0716	0.5833 ± 0.0578	0.5540 ± 0.0580	0.5000 ± 0.0613	0.6301 ± 0.0560	0.6383 ± 0.0614	0.6416 ± 0.0619	0.6154 ± 0.0797	0.7139 ± 0.0745	0.5977 ± 0.0614	0.6707 ± 0.0629
*AD*	0.7060 ± 0.0561	0.6077 ± 0.0495	0.6056 ± 0.0556	0.5812 ± 0.0614	0.6186 ± 0.0529	0.6265 ± 0.0500	0.6492 ± 0.0528	0.6008 ± 0.0631	0.7524 ± 0.0723	0.6262 ± 0.0457	0.6887 ± 0.0556
*CUD*	0.7634 ± 0.0484	0.6269 ± 0.0561	0.5963 ± 0.0501	0.5245 ± 0.0533	0.6761 ± 0.0474	0.6711 ± 0.0486	0.7003 ± 0.0578	0.6453 ± 0.0528	0.7659 ± 0.0624	0.6244 ± 0.0483	0.7253 ± 0.0540

**Table 7:** Mean (± SD) percentage bias and SSIM for low-dose participants between model prediction and ground truth for ROIs

Low-dose participants	Frontal	Insula	Hippocampus	Amygdala	Occipital	Parietal	Precuneus	Putamen	Pallidum	Temporal	Ant. Cingulate
Bias	0.94%±5.89%	3.42%±4.35%	3.20%±7.30%	5.02%±7.45%	-2.93%±5.53%	-2.80%±6.76%	-1.69%±6.40%	1.19%±6.22%	0.29%±7.32%	1.41%±4.96%	4.54%±5.62%
SSIM	0.5642 ± 0.0426	0.4392 ± 0.0407	0.4365 ± 0.0607	0.3998 ± 0.0617	0.4967 ± 0.0340	0.5069 ± 0.0166	0.4951 ± 0.0445	0.4606 ± 0.0564	0.5843 ± 0.0738	0.4652 ± 0.0439	0.5325 ± 0.0600

## Discussion

**Fig. 6:**
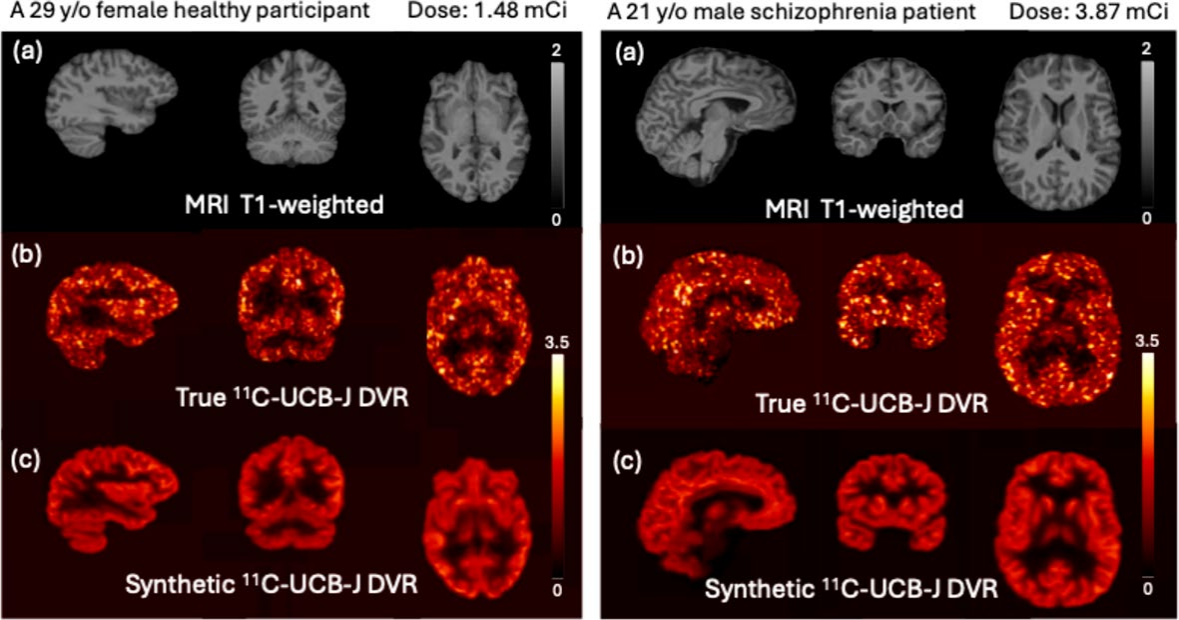
Two sample results from low-dose participants. (**a**) T1-weighted MRI, input of the model; (**b**) [^11^C]UCB-J PET DVR image, ground truth; (**c**) Synthetic [^11^C]UCB-J DVR image, output of the model

By generating synthetic [^11^C]UCB-J images, we developed a cost-efficient and accessible model to provide crucial information on the underlying pathology for certain neurological disorders without subjecting patients to additional radiation exposure. We proposed a method to enhance functional and metabolic insights by synthesizing images, with the aim of revealing more information from MRI scans. Additionally, in the case of low-dose participants, the model exhibits a notable capacity for noise reduction, indicating a potential for improved image quality and safety. As two sample studies shown in Fig. 6, participants with very low-dose injections have noisy PET images. In contrast, the generated images from MRI is of better quality and could potentially supplement an additional piece of diagnostic information, particularly in the situation where the real scans are of unacceptable quality.

We tested the performance of the model across diseases representing varying metabolic activities and demonstrated the feasibility of generating synthetic [^11^C]UCB-J PET images based on T1-weighted MR images with promising prediction accuracy for participants with SZ, AD, CUD, and healthy people. On the one hand, including diagnostic information during training could be a possible investigation direction. On the other hand, when the diagnostic information may not be readily available during the initial evaluation of new patients, [^11^C]UCB-J image prediction from MRI may be useful in monitoring disease progression and evaluating response to therapy for research use, especially for institutions where [^11^C]UCB-J tracers are not accessible. In addition, we noticed that among the diagnostic groups, the AD participants have the lowest prediction accuracy. Structural MRI reflects that AD can lead to significant brain atrophy [39] and studies [33, 40] also reported significantly reduced SV2A levels in many brain regions. AD presents distinct neurodegenerative patterns compared to SZ and CUD and the brain structure and image voxel values of AD participants is the most different from that of healthy participants compared to SZ and CUD. Our dataset is highly imbalanced, with a majority of participants being healthy controls. Consequently, the model may exhibit bias toward healthy individuals. Although the model demonstrates some degree of generalization ability, as indicated in Table 4, increasing the number of AD datasets to create a more balanced diagnostic ratio could directly address the low prediction accuracy for the AD group in future studies.

Our current work has several limitations. For the full-dose participants, we observed a reduction in noise but also a loss in resolution in the output images. This smoothing effect has been noted in medical imaging studies using convolution-based networks, which may result from up-sampling operations and pooling operations [41]. The use of L2 loss function tends to produce stable but blurry images rather than sharper details to achieve quantification accuracy [42]. Although these operations are highly efficient for high-level tasks like ours, they tend to bypass details in images and lead to a loss of resolution. To mitigate this effect, incorporating additional anatomical information and segmentation atlases might enhance spatial precision and delineation, potentially addressing this issue in future studies. Participants with data acquired using multiple imaging modalities, such as FDG PET, [^11^C]UCB-J PET, MRI, and DTI, could be used to train networks with multiple imaging inputs to improve the SV2A PET image prediction. Currently, our method offers a fast and robust approach with the available data. Notably, with the recent advancements, diffusion-based models have been utilized for medical image synthesis, primarily for super resolution, denoising or restoration tasks (e.g. low-count to high-count generation). Expanding this study to explore the possibility of developing DMs in cross-modality medical image translation, as well as the supporting training and inference techniques for better controllability and clinical reliability, would be a direction for future research. At present, while DMs can generate high-quality images, they suffer from hallucinations, and the reliance on extensive data and significant computational resources limits their accessibility for real-world applications [43, 44], especially for 3D imaging. In subsequent studies, increasing the training samples might enable the networks to capture more detailed variations in metabolic activity, potentially leading to sharper and more accurate image generation. It could also allow for the use of deeper or more complex high-fidelity image-to-image translation architectures, presenting a path for future advancements. Additionally, the stability of the model prediction is a direction for further investigation. The standard deviation of the percentage bias between the predicted images and the real images is a limitation of this study. Furthermore, we used cerebellum as the reference region to generate the DVR PET images for participants from all diagnostic groups, it would be interesting to explore whether using other reference regions (such as centrum semiovale) to generate the DVR images for training could possibly reduce the variability in our future study. Finally, downstream clinical analysis such as diagnosis and classification are yet to be done.

## Conclusion

We have demonstrated the feasibility to predict [^11^C]UCB-J images from T1-weighted MR images using a properly trained deep learning model. We concluded our model is able to learn the structural-functional relationship between the [^11^C]UCB-J images and the T1-weighted MR images at the individual level and produces desirable and robust synthetic [^11^C]UCB-J PET images. We provided a fast and accessible way of offering extra information on the pathology of certain neurological disorders. The utilization of 3D convolutional encoder-decoder networks for cross-modality translation from MRI to PET proves to be a viable approach applicable to different diagnostic types.

## Electronic supplementary material

Below is the link to the electronic supplementary material.

Supplementary Material 1

## Data Availability

The primary datasets analyzed during the current study are not publicly available due to matters of clinical data protection. Upon reasonable request, codes and secondary data may be available from the corresponding author.

## Abbreviations

SV2A: Synaptic vesicle glycoprotein 2 A
PET: Positron emission tomography
MRI: Magnetic resonance imaging
SZ: Schizophrenia
AD: Alzheimer’s disease
CUD: Cannabis use disorder
CNN: Convolutional neural network
BP_ND_: Non-displaceable binding potential
DVR: Distribution volume ratio
MSE: Mean squared error
SSIM: Structural similarity index measure
