# Supplementary material for: Generating synthetic brain PET images of synaptic density based on MR T1 images using deep learning
Source: EJNMMI Phys. 2025 Mar 31;12:30. doi: 10.1186/s40658-025-00744-5 (PMC11958861; doi:10.1186/s40658-025-00744-5)
Supplement: Supplementary file 1 — Supplementary Material 1 [file 40658_2025_744_MOESM1_ESM.docx]

**Supplementary Information**

**TABLE S1**: Details of T1-weighted MPRAGE.

| ***Scanner*** | ***N*** | ***TR / TE*** | ***Flip*** | ***In-plane*** | ***Thickness*** | ***Slices*** | ***FoV*** | ***Seconds*** |
| --- | --- | --- | --- | --- | --- | --- | --- | --- |
| PrismaFit | 41 | 2530 / 2.81 | 7 | 1.00 × 1.00 | 1.00 | 176 | 256 × 256 | 487 |
| PrismaFit | 20 | 2530 / 2.44 | 9 | 1.00 × 1.00 | 1.00 | 176 | 256 × 256 | 272 |
| PrismaFit | 15 | 2300 / 2.95 | 9 | 1.05 × 1.05 | 1.20 | 211 | 253 × 270 | 312 |
| PrismaFit | 2 | 2500 / 2.82 | 7 | 0.98 × 0.98 | 1.00 | 176 | 250 × 250 | 1092 |
| TIM Trio | 41 | 2500 / 2.78 | 7 | 0.98 × 0.98 | 1.00 | 176 | 250 × 250 | 1092 |
| TIM Trio | 37 | 2300 / 2.95 | 9 | 1.05 × 1.05 | 1.20 | 211 | 253 × 270 | 312 |
| TIM Trio | 1 | 1900 / 2.52 | 9 | 0.98 × 0.98 | 1.00 | 176 | 250 × 250 | 258 |
| TIM Trio | 1 | 2400 / 1.90 | 8 | 1.00 × 1.00 | 1.00 | 208 | 256 × 256 | 345 |
| TIM Trio | 2 | 2530 / 2.77 | 7 | 1.00 × 1.00 | 1.00 | 176 | 256 × 256 | 487 |
